# Insufficient Anthrax Lethal Toxin Neutralization Is Associated with Antibody Subclass and Domain Specificity in the Plasma of Anthrax-Vaccinated Individuals

**DOI:** 10.3390/microorganisms9061204

**Published:** 2021-06-02

**Authors:** Kenneth Smith, Lori Garman, Kathleen Norris, Jennifer Muther, Angie Duke, Renata J. M. Engler, Michael R. Nelson, Limone C. Collins, Christina Spooner, Carla Guthridge, Judith A. James

**Affiliations:** 1 Department of Arthritis and Clinical Immunology, Oklahoma Medical Research Foundation, 825 NE 13th St., Oklahoma City, OK 73104, USA; kathleen-norris@omrf.org (K.N.); jenny143m3@gmail.com (J.M.); angie-duke@omrf.org (A.D.); carla-guthridge@omrf.org (C.G.); 2 Department of Genes and Human Disease, Oklahoma Medical Research Foundation, 825 NE 13th St., Oklahoma City, OK 73104, USA; lori-garman@omrf.org; 3 Walter Reed National Military Medical Center, 8901 Wisconsin Ave, Bethesda, MD 20814, USA; renata.engler@gmail.com (R.J.M.E.); michael.r.nelson4.civ@mail.mil (M.R.N.); limone.c.collins.civ@mail.mil (L.C.C.); christina.e.spooner.civ@mail.mil (C.S.); 4 Department of Microbiology and Immunology, Oklahoma University Health Science Center, 940 Stanton L. Young Blvd, Oklahoma City, OK 73104, USA; 5 Departments of Medicine and Pathology, Oklahoma University Health Science Center, 1000 Stanton L. Young Blvd, Oklahoma City, OK 73104, USA

**Keywords:** anthrax, vaccine, antibody, subclass, domain, toxin

## Abstract

Anthrax vaccine adsorbed (AVA) is a significant line of defense against bioterrorist attack from *Bacillus anthracis* spores. However, in a subset of individuals, this vaccine may produce a suboptimal quantity of anti-protective antigen (PA), antibodies that are poorly neutralizing, and/or antibody titers that wane over time, necessitating annual boosters. To study individuals with such poor responses, we examine the properties of anti-PA in a subset of vaccinated individuals that make significant quantities of antibody but are still unable to neutralize toxin. In this cohort, characterized by poorly neutralizing antibody, we find that increased IgG4 to IgG1 subclass ratios, low antibody avidity, and insufficient antibody targeting domain 4 associate with improper neutralization. Thus, future vaccines and vaccination schedules should be formulated to improve these deficiencies.

## 1. Introduction

*Bacillus anthracis* is a Gram-positive, spore-forming bacterium responsible for anthrax infection that poses a great threat as an agent of bioterror [1]. Following inhalation and germination of spores, the bacilli produce lethal toxin (LT), composed of protective antigen (PA) and lethal factor (LF), as well as edema toxin (ET), composed of PA and edema factor (EF) [2]. PA has four defined protein domains: 1, 2, 3, and 4. PA binds to cell surface receptors via domain 4 and is then cleaved by furin-like proteases at a site within domain 1, yielding PA63 and the amino-terminal fragment domain 1A (PA20). PA63 then oligomerizes via domain 3 to form a pore in the surface of target host cells. After binding of LF or EF through domain 1′ (remaining on PA63) and endocytosis of the toxin/pore complex, the enzymatic toxins EF and LF are released into the cytosol.

Antibodies directed toward PA can effectively protect from the enzymatic activities of LF or EF in both in vitro and in vivo rabbit, non-human primate, and some murine models [3,4,5,6,7]. Thus, whole PA is the primary immunogen of Anthrax Vaccine Adsorbed (AVA), with smaller unquantified amounts of LF. In many well-controlled studies of AVA or PA vaccination, the plasma concentration of anti-PA IgG correlates strongly with the ability of immune plasma to neutralize LT in vitro and protect animals from *Bacillus anthracis* challenge [8,9,10]. However, after natural cutaneous anthrax infection, much of the early antibody response is focused toward LF as well as anti-PA [11,12]. Work in our lab and others has shown that the antibody response to the anthrax vaccine utilized in the United Kingdom (AVP, anthrax vaccine precipitated), which contains quantified amounts of LF (7.9 µg/mL PA, 1.9 µg/mL LF [13]), elicits higher neutralizing response as determined by in vitro lethal toxin neutralization assays than AVA likely due to invoking higher quantities of anti-LF [14,15].

As a basis for this study, we have previously determined in a large, real-world cohort that a portion of AVA-vaccinated personnel possess moderate or high anti-PA IgG concentrations, yet do not neutralize LT in vitro [16,17]. Of individuals in our cohort vaccinated 3 or more times with AVA and possessing at least 25 µg/mL plasma anti-PA IgG, almost one-fifth neutralize toxin no better than unvaccinated controls (286/1440, 19.9%) [16].

Thus, this study evaluates characteristics of the response to AVA vaccination of a cohort of individuals producing poorly neutralizing antibody. We considered several possible explanations for the poor neutralization of antibodies produced by this cohort. First, these individuals may make an antibody response dominated by a subclass that is poorly neutralizing. Not all IgG subclasses of anti-PA may be equally protective against anthrax exposure. IgG4, for example, is functionally monovalent [18] and engages Fc receptors differently than IgG1, which may influence its neutralization potential [19,20]. Second, these individuals may make antibodies with poor avidity. The presence of high avidity antibodies generated in response to vaccination generally indicates that a T-cell-dependent response and affinity maturation have occurred, but avidity may or may not affect function [21]. For example, in a study of anti-snake venom IgG in camels, neither titer nor avidity correlated with venom neutralization [22]. However, in a mouse model of *Escherichia coli* enterotoxin immunization, avidity increased concurrent with neutralization and titer [23]. Finally, the antibodies made by these individuals may not recognize epitopes critical for neutralization. It is readily apparent from monoclonal antibody studies, PA domain and epitope immunization studies, and B-cell epitope mapping, that the epitopes recognized by the antibody are crucial for efficient neutralization. For instance, monoclonal antibodies that bind PA domain 1A [24,25] or domain 3 [25] are unlikely to neutralize toxin. Antibodies binding to domain 4 often have potent neutralization, making recombinant domain 4 a viable vaccine antigen [26,27]. Thus, here we examined the subclass, avidity, and domain usage of anti-PA in individuals with poorly neutralizing antibody.

## 2. Materials and Methods

### 2.1. Collection of Human Blood Samples

AVA vaccinated individuals were enrolled with informed consent. Volunteers provided demographics (sex, age, race) and vaccination history (detailed in Table 1). Institutional Review Board approval was obtained from the Oklahoma Medical Research Foundation, University of Oklahoma Health Sciences Center, and Walter Reed National Military Medical Center. This work has been carried out in accordance with The Code of Ethics of the World Medical Association (Declaration of Helsinki). Plasma was collected and stored at ≤−20 °C until further use.

### 2.2. Anti-PA IgG (Total and Subclass) Concentration

High binding ELISA plates (Costar 3369, Corning, NY, USA) were coated with 1 µg/well of recombinant PA (List Biological Laboratories, Inc., Campbell, CA, USA), all washes were with PBS-Tween (0.05% Tween), and plates were blocked with 0.1% BSA in PBS. Standard reference serum AVR801 [28] (109.4 µg/mL anti-PA; Center for Disease Control and Prevention, Atlanta, GA, USA) was serially diluted 2-fold at a starting concentration of 2 µg/mL for total anti-PA. Similarly, purified myeloma IgG1 and IgG4 (Athens Research, Athens, GA, USA) directly coated on the plate starting at 20 µg/mL were used for subclass standard curves. Plasma samples were run at 1:200 and 1:400 dilutions in duplicate wells (samples with very high anti-PA occasionally required 1:800 dilution) for total anti-PA and at 1:100 and 1:200 for subclass quantification. Total IgG was detected with AP-conjugated goat anti-human IgG (Jackson Immunoresearch, West Grove, PA, USA) and PNPP (Sigma-Aldrich, St. Louis, MO, USA) and read at 410 nm. IgG1 and IgG4 anti-PA were detected with HRP-conjugated mouse anti-human IgG4 and IgG1 (Invitrogen, Eugene, OR, USA) and Super AquaBlue ELISA substrate (Invitrogen, Eugene, OR, USA) and read at 405 nm. The standard curves were fit with a 4-parameter curve fit in GraphPad Prism and the concentration of total anti-PA or subclasses in samples were interpolated from OD values. Each sample was analyzed on three independent runs; if the three runs did not agree within 50% of the average, samples were run for a fourth time.

### 2.3. LT Neutralization Activity (LTNA)

Recombinant PA and LF were purchased from List Biological (List Biological Laboratories Inc., Campbell, CA, USA). The ability of participant plasma to neutralize LT (LTNA) was adapted from the standard procedure from the CDC as previously described [29] using the J774.1 macrophage cell line (ATCC, Manassas, VA, USA). In brief, cells were plated overnight at 90,000 cells/well in a 96-well plate. Plasma samples were diluted 1:50 in culture medium, and then diluted for eight additional serial two-fold dilutions in dilution tubes, and then incubated with LT for 30 min at 37 °C (600 ng/mL of PA, 120 ng/mL of LF) before adding to cell cultures. Control wells included cells alone, cells with added PA only, LF only, or cells with PA and LF (LT). For quality control [30], rather than AVR801, a neutralizing fully human antibody (p6C01, [25]) was included for each assay, and plates in which p6C01 returned a value greater than one standard deviation from the average total p6C01 EC50 runs (*n* = 66) were not used. Toxin/serum mixtures were then incubated on the cells for 4 h at 37 °C. Viability was assessed by addition of MTT (Sigma-Aldrich, St. Louis, MO, USA) to each well for 2 h at 37 °C. The plates were then incubated at 37 °C overnight and read at 570 nm. ED50s were calculated using a four parameter (sigmoidal) curve fit. Each plasma sample was run at least three times and average ED50s were calculated.

### 2.4. Anti-PA IgG Avidity

The avidity index (AI) was calculated in a manner similar to previous studies [9]. Plates were coated with recombinant PA at 0.1 µg/mL. Sera were also diluted to 0.1 µg/mL anti-PA IgG and were allowed to bind to immobilized PA for two hours before elution with six concentrations of ammonium thiocyanate (0.15 to 4 M) for 20 min (Sigma). The amount of anti-PA remaining bound was detected in an anti-PA ELISA as above. A four parameter (sigmoidal) dissociation curve was generated for the percent maximum detected signal versus log of the NH_4_SCN concentration, and the avidity index (AI) is reported as the molar concentration of NH_4_SCN required to elute 50% of bound anti-PA IgG.

### 2.5. Recombinant Protective Antigen Domains and ELISA

cDNA sequences for PA domains 1A, 3, and 4 were generated by RT-PCR [31] and cloned into a pGEX-6P-1 vector (GE Healthcare, Pittsburgh, PA, USA). BL21 cells were transformed with PA/pGEX-6P-1 vectors. IPTG was added to the cultures to induce production of PA subunits at 16 °C. GST spin columns were used to purify the PA domains (Pierce; B-PER GST Fusion Protein Spin Purification Kit; Rockford, IL, USA). The proteins were then quantified by nanodrop. Individual domains were coated on high binding ELISA plates at 1 µg/well (as in Section 2.2) and compared to a standard of directly-coated recombinant human monoclonal IgG1 starting at 10 µg/mL. The detection antibody is the same as for total anti-PA (Section 2.2). Curve fitting with Prism is also as detailed for anti-PA above.

### 2.6. Statistical Analysis

Individuals with high LTNA (ED50 > 100) and low LTNA (ED50 < 100) were cohort-matched by race, sex, years since last vaccination, and number of vaccinations. All comparisons between such matched high LTNA and Low LTNA groups were analyzed using Wilcoxon Signed Rank Tests. All correlations were performed with Spearman’s two-tailed correlation test.

## 3. Results

### 3.1. Over Half of AVA Vaccinees Produce Insufficient Quantity and/or Quality of Anti-PA

Our cohort was comprised of 144 AVA vaccinees. While all of these individuals received the AVA priming series, this is a “real world” cohort of military personnel that did not, in general, follow the recommended dosing schedule. Collected vaccination history for each donor sample included the total number of AVA vaccinations and time since last vaccination, defined as the time interval between last boost and sample collection date.

Antibody quantity in all samples was measured by total anti-PA IgG ELISA and compared to the hypothesized, protective cutoff of 97.3 µg/mL from non-human primate challenge studies [9]. Although this value is from rhesus macaques and was determined at 1 month after third vaccine dose (7 months total) and cannot be related to protection in humans, we used this number as a comparator to our cohort. Thus, the majority of our cohort (54.2%) had serum anti-PA concentrations of less than 97.3 µg/mL. Of serum samples collected more than two years after last vaccination, 82.8% had less than 97.3 µg/mL of anti-PA, indicating that without yearly boosts serum anti-PA concentrations wane significantly.

**Figure 1:**
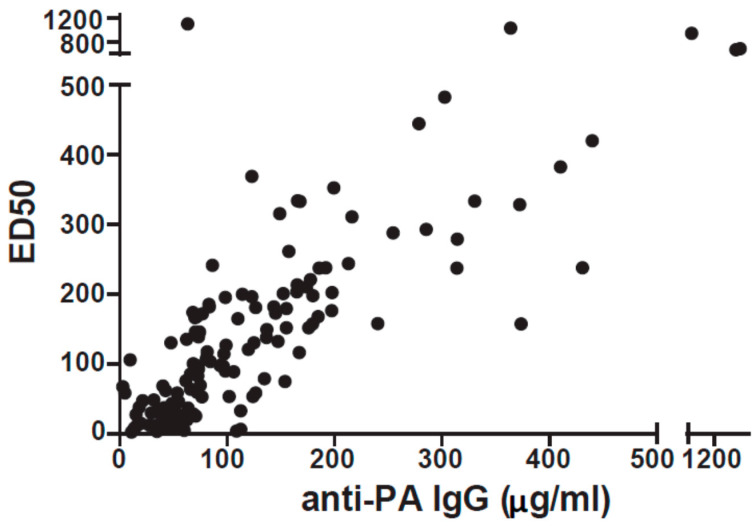
Anti-PA concentration vs. neutralizing potential (ED50) for 144 AVA vaccinees. A strong correlation between anti-PA IgG concentration and ED50 (*r_s_* = 0.82; *p_s_* < 0.0001) is shown (Spearman’s correlation).

Antibody quality was measured by lethal toxin neutralization assay and expressed as the effective dilution of plasma necessary to protect 50% of plated cells from death (ED50). An ED50 of 100 was chosen as the cutoff for neutralization, corresponding to a percent viability of 50% as previously published in reports by our lab [16]. Almost half of our cohort (47.9%) were poorly neutralizing (ED50 < 100), and almost a third (30.6%) were very poorly neutralizing (ED50 < 50). As expected, there was a strong correlation between anti-PA IgG concentration and ED50 (*r* = 0.82; *p* ≤ 0.0001, Figure 1), indicating that in general, higher anti-PA in the serum corresponds with greater neutralization capacity.

**Figure 2:**
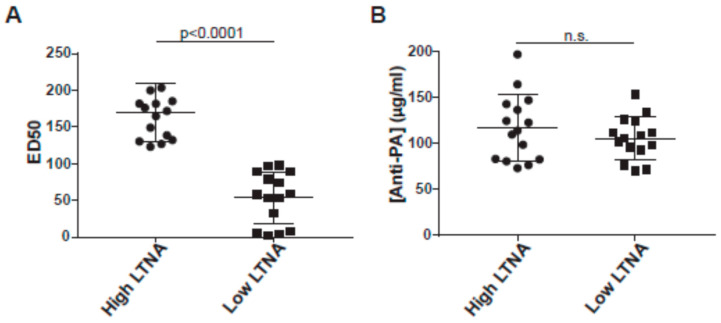
Characteristics of the high and low neutralizing matched cohort. (**A**) High neutralizers were defined as those subjects with ED50 higher than 100 and (**B**) were matched with low neutralizers, defined as those subjects with ED50 below 100, on anti-PA concentration. Data from high neutralizers are shown as circles, data from low neutralizers are shown as squares.

To isolate the effect of antibody quality from quantity, we chose to focus on 15 individuals with anti-PA levels greater than 70 µg/mL, but ED50 less than 100 (mean: 170). These 15 vaccinees, defined as low neutralizers, were matched with 15 high neutralizers who also had anti-PA levels greater than 70 µg/mL, but ED50 values of >100 (mean: 53.6) (details of the cohort in Table 1; similarly, see Supplementary Table S1 for a summary of the entire cohort). It was necessary to use a 70 µg/mL cutoff (rather than 97.3 µg/mL) to have sufficient high neutralizers for matching and it was a natural cutoff in our cohort (see Figure 1). Matching was based on number of vaccinations (tolerance: ±1 vaccination), years since last vaccination (largest difference is 7.2 months), and anti-PA concentrations (50% max difference, all between 70 and 200 µg/mL). Figure 2 shows the ED50 (A) and anti-PA concentrations (B) for the cohort and show that the cohort has approximately the same amount of anti-PA IgG but differ greatly in toxin neutralization capacity.

### 3.2. IgG4/IgG1 Ratios Are Associated with Neutralization

**Figure 3:**
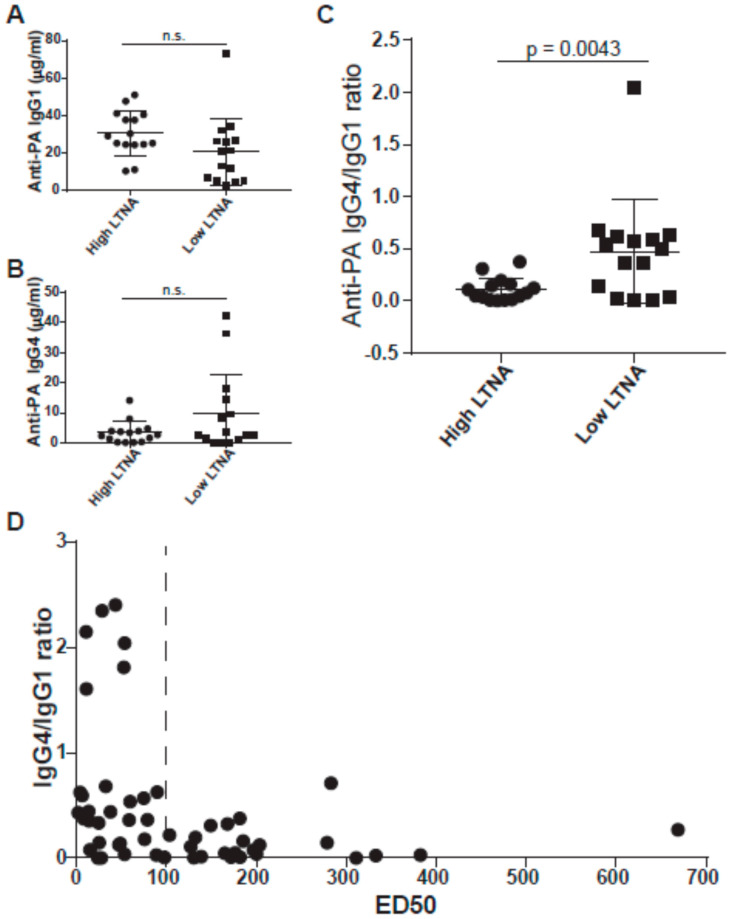
IgG subclass concentrations by ELISA in the matched cohort. (**A**) Anti-PA IgG1 concentrations show a non-significant higher trend in high neutralizers. (**B**) Anti-PA IgG4 concentrations show a non-significant higher trend in low neutralizers. (**C**) Low neutralizers have a significantly higher IgG4/IgG1 ratio. In panels A, B, and C, data from high neutralizers are shown as circles; data from low neutralizers are shown as squares. (**D**) When expanded from 30 individuals to 53 individuals including 23 that have received 8, 9, or 10 vaccinations, a striking subset of 6 individuals with IgG4/IgG1 ratio over 1.0 who are low neutralizers becomes evident.

We hypothesized that one important difference between the high and low neutralizers may be subclass distribution, particularly with respect to IgG4. Thus, we analyzed our matched cohort (*n* = 30) for anti-PA IgG subclass concentrations by ELISA. As shown in Figure 3A, the high neutralizers have slightly higher anti-PA IgG1 concentrations, but the difference is not significant. There was also no significant difference in the concentrations of anti-PA IgG4 (Figure 3B) between the two groups, but several individuals in the low neutralizer group had quite high levels of anti-PA IgG4. To examine the relationship between anti-PA IgG1 and IgG4 in each sample, we then expressed them as the ratio of anti-PA IgG4 to IgG1 (Figure 3C). Low neutralizers were found to have significantly higher IgG4 to IgG1 ratios (*p* = 0.0043).

Recent data from Quinn et al. utilizing different dosing schedules in an AVA clinical trial found increasing anti-PA IgG4 levels over 30 months past first vaccination (8-SQ, 6 subcutaneous doses plus two-yearly boosts) [32]. Therefore, we analyzed anti-PA IgG4 and IgG4/IgG1 ratios in samples from individuals receiving 8, 9, or 10 total AVA vaccinations (*n* = 23), as this timing is beyond 30 months since first vaccination. In this expanded cohort of 53 individuals, we see a striking feature (Figure 3D). All six vaccinees (over 10% of the cohort) with an IgG4/IgG1 ratio of over 1.5 had ED50′s less than 100. Thus, our findings indicate that a high anti-PA IgG4/IgG1 ratio of greater than 1.5 correlates with an ED50 < 100 and may explain poor neutralization in this cohort with a high number of AVA vaccinations. We also analyzed anti-PA IgG2 (data not shown), IgG3 (data not shown), IgA (Supplementary Figure S1A), and IgE (Supplementary Figure S1B), but found no significant differences between high and low neutralizers. As IgG4 has been linked to immune responses from allergen immunotherapy, we visualized the correlation between IgG4 and IgE anti-PA (Supplementary Figure S1C), but no correlation was found.

### 3.3. Anti-PA Avidity Is Associated with the Number of AVA Vaccinations

**Figure 4:**
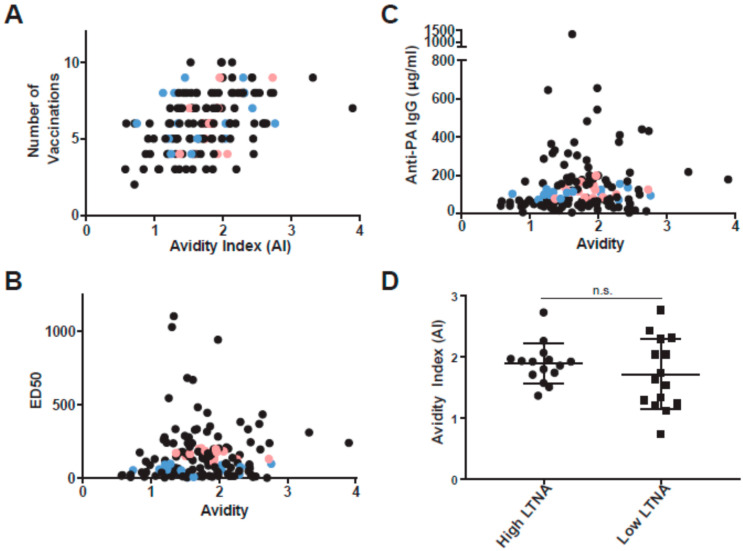
Antibody avidity by thiocyanate elution ELISA. (**A**) Avidity index plotted vs total number of vaccinations in all 144 individuals shows a positive Spearman’s correlation (*r_s_* = 0.475; *p_s_* < 0.0001). However, there is no correlation between avidity index and (**B**) ED50 (*r_s_* = 0.114; *p_s_* = 0.1761) or (**C**) anti-PA concentration (*r_s_* = 0.154; *p_s_* = 0.0667), In panels (**A**–**C**) the black dots are from the total cohort; from this cohort, data from high neutralizers from the matched cohort are shown in pink while data from low neutralizers are shown in blue. (**D**) In the matched cohort, several low neutralizers have lower avidity indexes than the high neutralizers, but there is no significant difference between the groups (data from high neutralizers are shown as circles, data from low neutralizers are shown as squares).

We also hypothesized that the avidity of the serum antibodies may be an important factor for proper neutralization. Utilizing thiocyanate elution ELISAs, we calculated the avidity index (AI) for each sample. We first compared AI to vaccination history and AI correlated significantly, although loosely, with total number of vaccinations in our total cohort (*n* = 144, Figure 4A). This finding confirms the basic immunological principle that as the humoral immune system is exposed to more antigen over time, the antibody response affinity matures. We then compared AI to quantity and quality of anti-PA. We found that AI was not correlated to LTNA ED50 (Figure 4B) or anti-PA IgG concentrations (Figure 4C) indicating that affinity maturation, in this case, does not associate with either quantity or neutralization capacity of anti-PA. Comparisons of AI in our matched cohort found no significant difference between high and low neutralizers (Figure 4D). However, we found that six of the low neutralizers had very low antibody avidities (below all high neutralizers). Thus, antibody avidity, like IgG4/IgG1 ratio may affect the quality of a subgroup of individual vaccinees.

### 3.4. Anti-Domain 4 Is Strongly Associated with Neutralization

**Figure 5:**
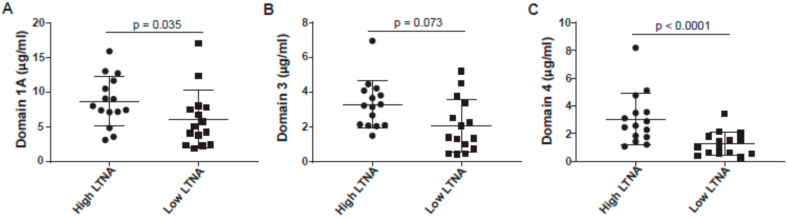
Anti-PA domain specificity by recombinant domain ELISA. (**A**) Anti-domain 1A and (**B**) anti-domain 3 concentrations show slightly significant or non-significant differences in concentration between high and low neutralizers, respectively. (**C**) The anti-domain 4 concentration difference is strongly significant with low neutralizers making less anti-domain 4 antibody. Data from high neutralizers are shown as circles; data from low neutralizers are shown as squares.

Responses directed toward certain domains of PA are necessary for proper toxin neutralization. Although domain 1A (PA-20) is highly immunogenic and dominates the antibody response to whole PA, antibodies to this domain are typically poorly neutralizing. In contrast, antibodies to domain 1′/2 (EF/LF binding) and domain 4 (receptor binding) are often highly neutralizing. We therefore explored domain specificity in our cohort as a potential measure affecting neutralization. Figure 5A,B show that the high neutralizers have more antibodies to domain 1A and 3, but barely or not significantly, respectively. However, we found that low neutralizers were significantly deficient in the amount of anti-domain 4 (Figure 5C). We were unable to test domain 2 by this method because of difficulties expressing this construct.

**Figure 6:**
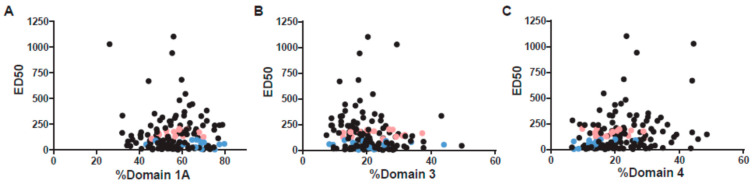
Anti-PA domain specificity in all 144 individuals. To account for large differences in total anti-PA IgG in the entire group of 144 individuals, domain concentrations were expressed as the percent of (**A**) anti-domain 1A and (**B**) anti-domain 3 with respect to the total of all three domains (1A + 3 + 4). Anti-domain 1A is not correlated with ED50 (*r_s_* = −0.034; *p_s_* = 0.688), anti-domain 3 shows a weak negative correlation (*r_s_* = −0.184; *p_s_* = 0.028). (**C**) The anti-domain 4 percentage shows a positive correlation with ED50 (*r_s_* = 0.230; *p_s_* = 0.0057). All comparisons were calculated using Spearman’s correlation. The black dots are from the total cohort; from this cohort, data from high neutralizers from the matched cohort are shown in pink; data from low neutralizers are shown in blue.

We also extended these domain studies to the full cohort of 144 by expressing binding to each domain as a percentage of the total of the 3 domains tested, thus correcting for the variation of total anti-PA in this cohort. We then compared percent binding to each domain with ED50. ED50 was not associated with domain 1A binding (Figure 6A) but was weakly and inversely correlated with percentage domain 3 binding (Figure 6B). In contrast, there is a weak, but significant, correlation between anti-domain 4 concentration and ED50 in the total group (Figure 6C). Thus, many individuals may have poor toxin neutralization because of insufficient targeting of the IgG response to domain 4.

## 4. Discussion

We have described three potential factors that could lead to poor LT neutralization after AVA vaccination: a high IgG4/IgG1 ratio, low antibody avidity, and a low concentration of anti-domain 4. To characterize our group of low neutralizers we assessed total anti-PA, ED50, age, total number of AVA vaccinations, and years post last boost (years between sample draw and last boost) and we indicate the relevance of each potential factor for each vaccinee (Table 2). For three of the 15 low neutralizers, we could find no likely impact of any of these potential factors, suggesting low domain 2 binding, which we could not measure, or another yet unexplored factor as an explanation for low ED50. It is also possible that two of these unexplained low neutralizers, “Vaccinee 1” and “Vaccinee 5”, have sufficient neutralization, as their ED50 values are near the cutoff of 100 (89.0 and 97.7 respectively).

In well-controlled trials of human vaccinees and animal studies, anti-PA IgG concentration explains over 90% of variation in LTNA [8,9,10]. However, many individuals receiving AVA are not enrolled in clinical trials; instead, real-world vaccinees are much more likely to receive doses when sick, deviate from the recommended dosing schedule, or miss doses entirely; particularly in a military setting where vaccinations are given at deployment. We have demonstrated repeatedly that in a real-world cohort, anti-PA IgG concentrations predict LTNA, but with a lower level of confidence than in well-controlled trials [16,17]. Here again, we show that a large percentage of individuals (54.2%) produce less than the 97.3 µg/mL comparator from non-human primate studies. Most importantly, we find here that the anti-PA generated by individuals with low toxin neutralization capacity generally demonstrates one or more of the following characteristics: a higher IgG4 to IgG1 ratio, a lower avidity for PA, and/or decreased binding to PA domain 4.

It is important to note that even vaccinees with low levels of anti-PA or poorly neutralizing antibody may still be protected in the case of exposure to anthrax spores. We have shown that vaccinees have memory B cells, which are capable of producing anti-PA upon challenge [33]. Furthermore, protection in vivo may be conferred by antibody functions not measured by the in vitro toxin neutralization assay such as opsonization and complement activation. Finally, although our total AVA cohort has very low levels of anti-LF [14], it may be synergistic with anti-PA in providing toxin neutralization. We have also shown that anti-LF levels are typically exceedingly low in AVA vaccinees. We did verify the anti-LF levels in our cohort, there was no difference between high and low groups; two individuals in each group had measurable anti-LF at a 1:100 dilution.

Unsurprisingly, increased numbers of vaccinations are associated with IgG4 skewing, particularly with the 1970 licensed priming series (0, 0.5, 1, 6, 12, and 18 months), with or without the 0.5-month dose [32] as received by all the donors in the cohort analyzed here. Our finding of IgG4 skewing was not completely unexpected; the aluminum hydroxide adjuvant present in AVA promotes a Th2 driven humoral response [34]. Th2 cytokines (particularly IL-4 and IL-13) [35], along with IL-10 and IL-21 derived from regulatory T cells in response to frequent exposure to low concentrations of antigen, may preferentially drive IgG4 production [18]. Th2 responses may also promote production of IgE, although it has been shown that ‘healthy’ Th2 responses can produce IgG1 and IgG4 in the absence of IgE [36]. Our lab has also shown that individuals showing large local reactions to AVA had increased anti-PA IgE [37], although here, anti-PA IgE was not correlated to anti-PA IgG4. The crucial question, then, is how anti-PA IgG4 contributes to poor neutralization. We can hypothesize that poor avidity for PA due to IgG4 being functionally monovalent, differences in Fc engagement, and the inability of IgG4 to efficiently form large complexes to otherwise block proper toxin formation could all contribute. Elucidating these mechanisms will require further testing.

The avidity of antibody toward its antigen is controlled by multiple factors. Briefly, the germinal center (GC) reaction selects the highest-affinity B cell clones by the combination of two factors related to antigen availability: T cell help is restricted to the high-affinity B cells most capable of antigen uptake and presentation [38], and circulating antibody entering GCs competes with GC B cells for antigen in an affinity-dependent manner [39]. These mechanisms are consistent with the association of avidity index with total number of vaccinations. Each subsequent dose of AVA allows for not only additional GC and rounds of affinity maturation, but an opportunity for previously-produced high-affinity antibody to compete with newly-activated naïve B cell clones. Although it is often assumed that avidity of antibody for antigen influences its efficacy, this is not always the case. Especially in the case of toxin neutralization, the overall affinity may not be as important as for example, off-rate, recently shown for ricin antibodies [40], or targeting the most efficient epitope.

The difference we observed in high LTNA and low LTNA individuals in PA domain 4 binding (Figure 5C) adds to the growing amount of evidence that antibodies directed toward domain 4, the receptor binding site, are likely to be neutralizing. Immunization of mice with PA in which domain 4 has been removed shows a decrease in protection relative to mice immunized with whole PA [31,41]; furthermore, all mice immunized with any fraction of PA which contained domain 4 were protected from spore challenge [31]. In addition, multiple monoclonal antibodies directed against domain 4 protect against anthrax toxin or spore challenge [42,43,44]. Not all studies, however, support the efficacy of domain 4 immunization, including a study in which mice immunized with PA domain 4 were less likely generate LT-neutralizing antibody than those immunized with whole PA [45].

While the current practice of AVA immunization, if followed correctly, produces measurable levels of toxin-neutralizing anti-PA IgG in most vaccinated individuals, a significant number have insufficient quantity or quality of antibody. While not implying direct causation by any particular factor, here we demonstrate that antibodies of lower avidity, higher IgG4/IgG1 subclass ratios, and limited domain 4 binding associate with ineffective neutralization of lethal toxin. Future studies are needed to investigate possible mechanisms responsible for this outcome. Further, this study is limited by studying only donors vaccinated with AVA. Nonetheless, our findings present a potential catch-22 of the AVA schedule. In vaccinating repeatedly to maintain sufficient serum anti-PA we induce B cells to produce more anti-PA and with higher avidity. Yet at the same time, we stimulate the skewing of antibody production to less-effective subclasses. Future vaccination strategies, perhaps utilizing subunits, adjuvants and/or LF to elicit a more complete and IgG1-dominated response may yield more effective, long-term antibody responses.

## Figures and Tables

**Table 1:** Demographics and vaccination information summary of 15 high and 15 low neutralizers. For gender M refers to male; for race A refers to Asian, AA to African American, and AI to American Indian. Anti-PA values are in µg/mL.

	High LTNA	Low LTNA
**Gender:**		
M (%)	73.0%	100%
**Race:**		
European American (%)	93.3% (1 A)	86.7% (1 AA, 1 AI)
**Age at collection:**		
Average (SEM)	31.7 (2.29)	31.1 (1.37)
Median	31	31
Range	20–47	22–40
**Number of vaccinations:**		
Average (SEM)	6.33 (0.48)	6.4 (0.43)
Median	6	6
Range	4–9	4–9
**Years since last vaccination:**		
Average (SEM)	1.12 (0.27)	1.09 (0.28)
Median	0.88	0.73
Range	0.18–3.67	0.09–3.57
**Anti-PA:**		
Average (SEM)	117.2 (9.35)	105.9 (6.01)
Median	114.3	106.2
Range	73.37–197.4	70.5–154.0

**Table 2:** Possible reasons for low neutralization capacity for each vaccinee in the low LTNA group. The boxes colored yellow indicate a possible reason using the cutoffs of (>0.5 for IgG4/IgG1 ratio, <1.5 for AI, and <1.1 for anti-domain 4). These cutoffs correspond to exceeding the maximum value of the high neutralizers (IgG4/IgG1 ratio) or minimum value of the high neutralizers (AI and anti-domain 4).

Donor	Anti-PA (µg/mL)	ED50	Age	Total # of AVA Vaccinations	Years Post Last Boost	Possible Reason for Low LTNA		
						High IgG4/IgG1 Ratio (>0.5)	Low Avidity (<1.5 AI)	Low Anti- Domain 4 (<1.1 µg/mL)
Vaccinee 1	106.2	89.0	28	5	0.79	0.03	2.04	2.14
Vaccinee 2	108.8	3.9	23	4	0.56	0.62	1.54	0.55
Vaccinee 3	126.4	58.4	33	4	3.57	0.36	1.24	0.61
Vaccinee 4	112.6	6.4	25	5	0.21	0.59	1.63	0.29
Vaccinee 5	93.3	97.7	30	6	2.42	0.01	2.76	1.81
Vaccinee 6	134.8	78.8	32	7	1.89	0.36	2.43	0.95
Vaccinee 7	124.0	53.5	40	6	0.18	2.04	2.04	1.50
Vaccinee 8	98.2	89.7	22	5	0.14	0.63	1.21	1.42
Vaccinee 9	102.0	53.5	31	6	0.13	0.04	0.74	0.64
Vaccinee 10	112.5	32.6	37	6	0.09	0.68	1.34	1.00
Vaccinee 11	96.4	96.9	30	8	0.73	0.01	1.29	2.04
Vaccinee 12	72.0	59.5	39	8	1.60	0.54	1.12	0.42
Vaccinee 13	70.5	7.3	33	9	1.38	0.15	2.30	1.55
Vaccinee 14	154.0	74.7	34	8	0.36	0.57	2.32	3.42
Vaccinee 15	76.5	2.2	30	9	2.37	0.50	1.74	0.55

## Data Availability

The data presented in this study are available on request from the corresponding author. The data are not publicly available due to limitation within the nature of consent.

## Supplementary Materials

The following are available online at https://www.mdpi.com/article/10.3390/microorganisms9061204/s1, Table S1: Demographics and vaccination information summary of 144 AVA vaccinees. Figure S1: Determination of IgA and IgE concentrations by ELISA in the matched cohort.
