# Supplementary material for: Insufficient Anthrax Lethal Toxin Neutralization Is Associated with Antibody Subclass and Domain Specificity in the Plasma of Anthrax-Vaccinated Individuals
Source: Microorganisms. 2021 Jun 2;9(6):1204. doi: 10.3390/microorganisms9061204 (PMC8229884; doi:10.3390/microorganisms9061204)
Supplement: Supplementary file 1 [file microorganisms-09-01204-s001.zip › Sup_table_1_052421.pdf]

	Total Cohort
**Gender:**	
M (%)	91%
**Race:**	
European American (%)	[illegible]% (7 AA, 3 H, 1 A, 3 Mixed)
**Age at collection:**	
Average (SEM)	30.4 (0.58)
Median	28.5
Range	20-48
**Number of vaccinations:**	
Average (SEM)	6.15 (0.16)
Median	6
Range	2-10
**Years since last vaccination:**	
Average (SEM)	1.20 (0.094)
Median	0.86
Range	0.09-6.25
**Anti-PA:**	
Average (SEM)	142.1 (15.5)
Median	83.2
Range	2.9-1388
**LTNA (ED50):**	
Average (SEM)	155.2 (15.1)
Median	107.2
Range	2.0-1103
