# Supplementary figures and images for: Insufficient Anthrax Lethal Toxin Neutralization Is Associated with Antibody Subclass and Domain Specificity in the Plasma of Anthrax-Vaccinated Individuals
Source: Microorganisms. 2021 Jun 2;9(6):1204. doi: 10.3390/microorganisms9061204 (PMC8229884; doi:10.3390/microorganisms9061204)

# B

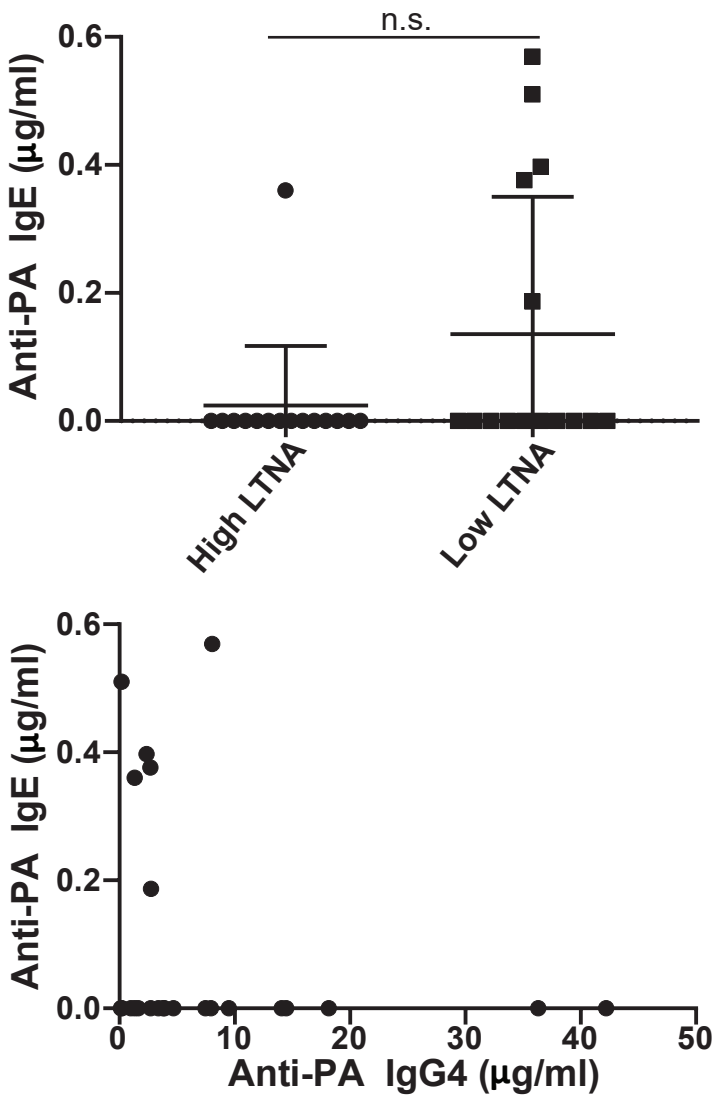

Supplement: Supplementary file 1 [file microorganisms-09-01204-s001.zip › Sup_fig1_new_042020.pdf]
